# Parents’ perceptions of functional electrical stimulation as an upper limb intervention for young children with hemiparesis: qualitative interviews with mothers

**DOI:** 10.1186/s12887-022-03403-1

**Published:** 2022-06-15

**Authors:** Emma Swaffield, Jaynie F. Yang, Patricia Manns, Katherine Chan, Kristin E. Musselman

**Affiliations:** 1 Lyndhurst Centre, KITE-Toronto Rehabilitation Institute, University Health Network, Toronto, Canada; 2 Dept. of Physical Therapy, Temerty Faculty of Medicine, University of Toronto, Toronto, Canada; 3 Dept. of Physical Therapy, Faculty of Rehabilitation Medicine, University of Alberta, Edmonton, Canada; 4 Neuroscience and Mental Health Institute, University of Alberta, Edmonton, Canada; 5 Rehabilitation Sciences Institute, Temerty Faculty of Medicine, University of Toronto, Toronto, Canada

**Keywords:** Perinatal stroke, Cerebral palsy, Functional electrical stimulation, Upper extremity, Qualitative research

## Abstract

**Background/objective:**

To explore parents’ perceptions of an upper extremity (UE) intervention using functional electrical stimulation (FES) for young children with hemiparesis.

**Methods:**

Parents of children aged 3–6 years with a history of perinatal stroke, impaired UE function, and participation in a 12-week FES intervention delivered at a hospital were included in this exploratory qualitative study. Nine mothers participated in a semi-structured interview < 1 week after their child completed the FES intervention (MyndMove®, MyndTec Inc.) targeting the hemiparetic UE. Open-ended questions queried parents’ goals, perceived benefits, and challenges of the FES intervention. Interviews were audio recorded and transcribed verbatim. Qualitative conventional content analysis was used to analyze the transcripts.

**Results:**

Five themes were identified. 1) Parents’ expectations for the FES intervention. Mothers described setting functional, exploratory, and realistic goals, yet feeling initial apprehension towards FES. 2) Perceived improvement. Physical, functional, and psychological improvements were observed with FES; however, there was still room for improvement. 3) Factors influencing the FES intervention. Program structure, therapist factors, and child factors influenced perceived success. 4) Lack of access to intensive therapy. Mothers noted that FES is not provided in mainstream therapy; however, they wanted access to FES outside of the study. They also highlighted socioeconomic challenges to accessing FES. 5) Strategies to facilitate participation. The mothers provided suggestions for program structure and delivery, and session delivery.

**Conclusions:**

Mothers perceived the FES intervention to have physical, functional and psychological benefits for their children. Interest in continuing with FES highlights a need to improve access to this therapy for young children.

## Background

Perinatal stroke is the most common form of childhood stroke [1, 2], often leading to abnormal sensorimotor development. More than half of children who sustain a perinatal stroke are diagnosed with unilateral cerebral palsy (CP) [3, 4]. These children present with spasticity, impaired sensation, and paresis, leading to reduced function [5], with the upper extremity (UE) typically more affected than the lower extremity [6]. Lack of function in the UE has a large impact on a child’s activities of daily living [7], quality of life [8], and self-esteem [9]. Thus, considering therapies that target the UE is important to address both physical and emotional dimensions of living with unilateral CP.

One intervention for the UE that is widely recognized as effective for adults living with hemiplegia is functional electrical stimulation (FES). FES is a therapy that incorporates principles of neuroplasticity and motor learning by using repetitive, task specific, and intensive practice [10]. FES involves the combination of functional movement with the appropriately timed application of a low-level electrical current to the skin overlying muscles or nerves in order to facilitate muscular contraction [11]. In adults with stroke, therapy involving motor level electrical stimulation is associated with improved muscle strength, increased range of motion, decreased spasticity, and improved UE activity and function [12–15]. As such, FES is included in best practice guidelines for management of the UE post-stroke [16, 17]; however, there has been little investigation into its application in children for improving UE function [18–21]. Moreover, the use of FES in pediatric clinical practice is likely limited. A recent survey on the use of FES by physical therapists revealed the perception that FES is contraindicated or inappropriate for pediatric populations [22]. Therapists also indicated that the strength of the evidence supporting FES influenced their use of this intervention [22]. Hence, more investigation into the feasibility and efficacy of FES for the UE in pediatric populations is needed to inform future use of this therapy.

The meaningfulness of an intervention, or whether its outcomes matter to the participating family, is another critical construct to investigate prior to clinical implementation [23]. Meaningfulness may be gauged through questionnaires [18, 21] or interviews [18, 24, 25] that query the perceptions and experiences of the child and/or parents. Parents are typically heavily involved in their child’s day-to-day life and have the potential to inform our understanding of the meaningfulness and perceived impact of an intervention. For example, our research team investigated the use of FES in a two-year-old child with perinatal stroke [18]. Through a parent questionnaire and semi-structured interview with the mother, we learned that the mother perceived the FES therapy as very worthwhile, but adhering to the high frequency of sessions (i.e. five days/week) was challenging [18]. Thoroughly understanding parents’ experiences will inform future research and clinical applications of UE FES for young children living with hemiplegia. In an effort to build upon our prior work, the purpose of this study was to explore parents’ perspectives of a UE FES intervention for young children with hemiparesis due to perinatal stroke.

## Methods

### Setting and participants

This qualitative descriptive study was part of a pilot study examining the efficacy of a FES intervention for improving UE function in children with perinatal stroke (ClinicalTrials.gov Identifier: NCT02975180). Ethical approval was obtained from the Research Ethics Board at the University Health Network, the University of Alberta, the Hospital for Sick Children and Holland Bloorview Kids Rehabilitation Hospital in Toronto, ON. All study activities were performed in accordance with the guidelines and regulations of the ethics boards. Written informed consent was obtained from a parent prior to study participation for all participants.

Parents of children that participated in the 12-week FES intervention for the UE were recruited for this study. To recruit participants for the pilot study, recruitment flyers were posted in children’s hospitals and pediatric therapy clinics in two cities, as well as distributed amongst a parent-led network for families with cerebral palsy. In order to be eligible, children had to meet the following criteria: 1) aged 3–6 years, 2) experienced a unilateral perinatal stroke with focal brain injury shown on an MRI, 3) have impaired UE function (i.e. levels II, III or IV on the Manual Ability Classification System) [26], and 4) able to sit unsupported for more than 5 minutes. Children were excluded from the study if they had any other disease, injury or condition that affected their UE motor function (e.g. contractures of the hand), an implanted electronic device, any surgical hardware in the UE, a history of epilepsy, a skin rash or wound at an electrode site, or if they had received a botulinum toxin to the UE in the past 6 months.

### Structure and components of FES intervention

The UE sessions were completed at the Lyndhurst Centre-University Health Network in Toronto or at the University of Alberta. Children were randomized into either the FES intervention group or the conventional UE training group (control) using a matched pairs design. Both groups were offered three one-hour sessions per week over 12 weeks, for a total of 36 hours of therapy targeting the hemiparetic UE. Participants were able to complete two sessions in one day with a minimum one-hour break between sessions to facilitate participation. For both groups, functional and age appropriate tasks that corresponded with the child’s goals and abilities were selected, and the child was encouraged to complete multiple repetitions of these movement patterns while playing. This session structure is in accordance with principles of neuroplasticity, such as specificity, salience, and repetition [10]. Approximately half of the one-hour session targeted unilateral UE activities with the affected arm, and the other half involved the use of both hands for bimanual tasks. The intervention sessions were fully supervised and administered by a registered physical or occupational therapist on a one-to-one basis. While practicing the functional tasks, the FES group received electrical stimulation using the MyndMove® FES system (MyndTec Inc., Mississauga, ON), while the conventional UE group did not. This eight-channel device allowed the therapist to select from 17 different reaching and grasping protocols as appropriate for the functional task being practiced. Families randomized into the control group were given the option to participate in the FES intervention once the follow-up period for the control intervention was complete.

### Data collection

For each child who received FES treatment, one parent participated in a semi-structured interview within one week of intervention completion. The interview was completed either over the phone (*n* = 3) or in-person at the Lyndhurst Centre (*n* = 6) by a researcher and physical therapist with qualitative research experience (KEM). The semi-structured interview guide consisted of open-ended questions exploring the family’s experience with FES (Table 1). Semi-structured interviews were chosen to enable a flexible and comprehensive exploration of the parent’s perceptions of FES for their child [27]. Immediately following each interview, the interviewer recorded her impressions of each interview and any patterns emerging across interviews (i.e. reflexive journaling). Interviews were audio-recorded and transcribed verbatim. Any identifiable information was removed to maintain participant privacy.

**Table 1:** Semi-structured interview guide

Interview with Parents of Children Participating in a Functional Electrical Stimulation (FES) Intervention	
We would like to hear about your/your child’s experience with FES (functional electrical stimulation). 1. What went well? 2. What was challenging? 3. Did you notice changes in your child’s abilities or play habits over the FES trial? If yes, please describe. 4. What was your/your child’s reaction to using FES? Did your/their reaction change over the length of the trial? 5. What were your goals for participation? Do you think you reached those goals? 6. If you were to discuss this trial with a parent/child who was thinking about participating, what would you tell them about using FES? 7. Do you have suggestions for things we could do differently, if we were to offer this program again with another child?	

### Data analysis

Following transcription, a qualitative conventional content analysis was applied, which is an inductive approach to describing phenomena for which existing theory or research is minimal [28]. Initially, two researchers with a background in physical therapy (ES and KEM) reviewed the first three transcripts and noted ideas and terms that reflected the content of the text. The researchers then discussed their notes, creating a coding scheme and identifying potential themes. For example, across the three transcripts mothers discussed the improvements they observed in their children, such as reduced tone, increased range of motion, and improvements in feeding and play. In this case, a code was created for each improvement and similar codes were grouped into potential sub-themes (e.g. physical and functional) and themes (e.g. Perceived improvement) (see Table 2). The coding scheme was then applied to the remaining interviews by the same two researchers, with discrepancies addressed, new codes created, and new sub-themes and themes developed through discussion. A third researcher (KC) then imported the transcripts and codes into NVivo 12 (QSR International), applying the codes to the transcripts and reviewing the coding scheme and previously identified themes throughout this process. The imported transcripts and codes were reviewed by the researchers, and the larger themes, relationship between themes, and sub-themes were discussed and documented. Several steps were taken to ensure trustworthiness of the interview data; specifically, the interviewer (KEM) had considerable experience conducting semi-structured interviews and content expertise in neurorehabilitation, and she maintained a reflective journal throughout data collection [29].

**Table 2:** Themes, sub-themes and codes

Themes	Sub-themes	Codes
1) Parents’ expectations for the FES intervention	a) Functional, exploratory, and realistic goals	- Functional goals - Explore potential of FES - Value of research - Complete recovery not expected - Expectations on retention - Goal of maintenance
	b) Initial apprehension towards FES	- Concerned about child’s tolerance of stimulation - Potential impact of sensory challenges
2) Perceived improvement	a) Physical	- Reduced tone - Increased range of motion - Increased awareness and sensation - Decreased range of motion - Rate of physical improvement
	b) Functional	- Improvements in dressing - Improvements in feeding - Improvements in play - New uses of affected arm - Increased frequency of unprompted arm use - Improved movement planning
	c) Psychological	- Improved confidence - Pride in new abilities
	d) Still room for improvement	- Progress made - Potential for more improvement
3) Factors influencing the FES intervention	a) Structure	- Flexibility of structure is beneficial - Volume is beneficial - Volume is a challenge - Travel time is a challenge - Therapy worth travel time
	b) Therapist factors	- Flexibility of therapist - Individualization in sessions - Rapport with child
	c) Child factors	- Characteristics of child that facilitated FES - Child not always cooperative - Child needs to be comfortable with therapist - Tolerance of electrical stimulation - Experiences with prior therapy facilitated FES
4) Lack of access to intensive therapy	a) Not provided in mainstream therapy	- Lack of public services - Lack of regular therapy - Different from conventional therapy
	b) Wanting access to FES outside of the study	- Home FES - Availability of FES in private sector - Availability of FES for adults versus children - Role as advocate for FES
	c) Socioeconomic challenges to access	- Frequency of sessions - Facilitators of participation - Social class - Cost of FES
5) Strategies to facilitate participation	a) Program structure and delivery	- Preference for FES at home - Preference for FES outside of home - Intensive camps - FES with other therapies - Child-friendly environments - Assistance with transportation
	b) Session delivery	- Distraction - Strategies to increase familiarization with FES - Incentives - Warm-up - Presence of parent at session

## Results

### Participant characteristics

Nine of the ten families who participated in the FES intervention agreed to participate in this qualitative study. One family withdrew after 14 FES sessions because the child disliked the electrical stimulation; this family declined to participate in the semi-structured interview. The remaining nine children each completed 36 sessions of the FES intervention (two completed the intervention at the University of Alberta). The median (range) length of time required to complete 36 hours of training was 15.6 (12.6–22.0) weeks for the nine children. One child experienced an illness in the middle of the training period that prevented participation for several weeks; it took this child 17.6 weeks to complete 36 training sessions. Another child’s training period was interrupted by a multi-week research shutdown due to the covid-19 pandemic. This child took 22.0 weeks to complete 36 training sessions. The remaining seven children completed the training period in 14.9 (12.6–16.3) weeks. The median (range) age of the children was 3.7 (3.1–6.4) years. Six child participants were male, and one male participant was also diagnosed with autism spectrum disorder. Three child participants completed the FES intervention after completing the control intervention. In all cases, the child participants’ mothers participated in the semi-structured interview. The median (range) length of the interviews was 25.4 (18.2–37.7) minutes.

### Themes and categories

**Fig. 1:**
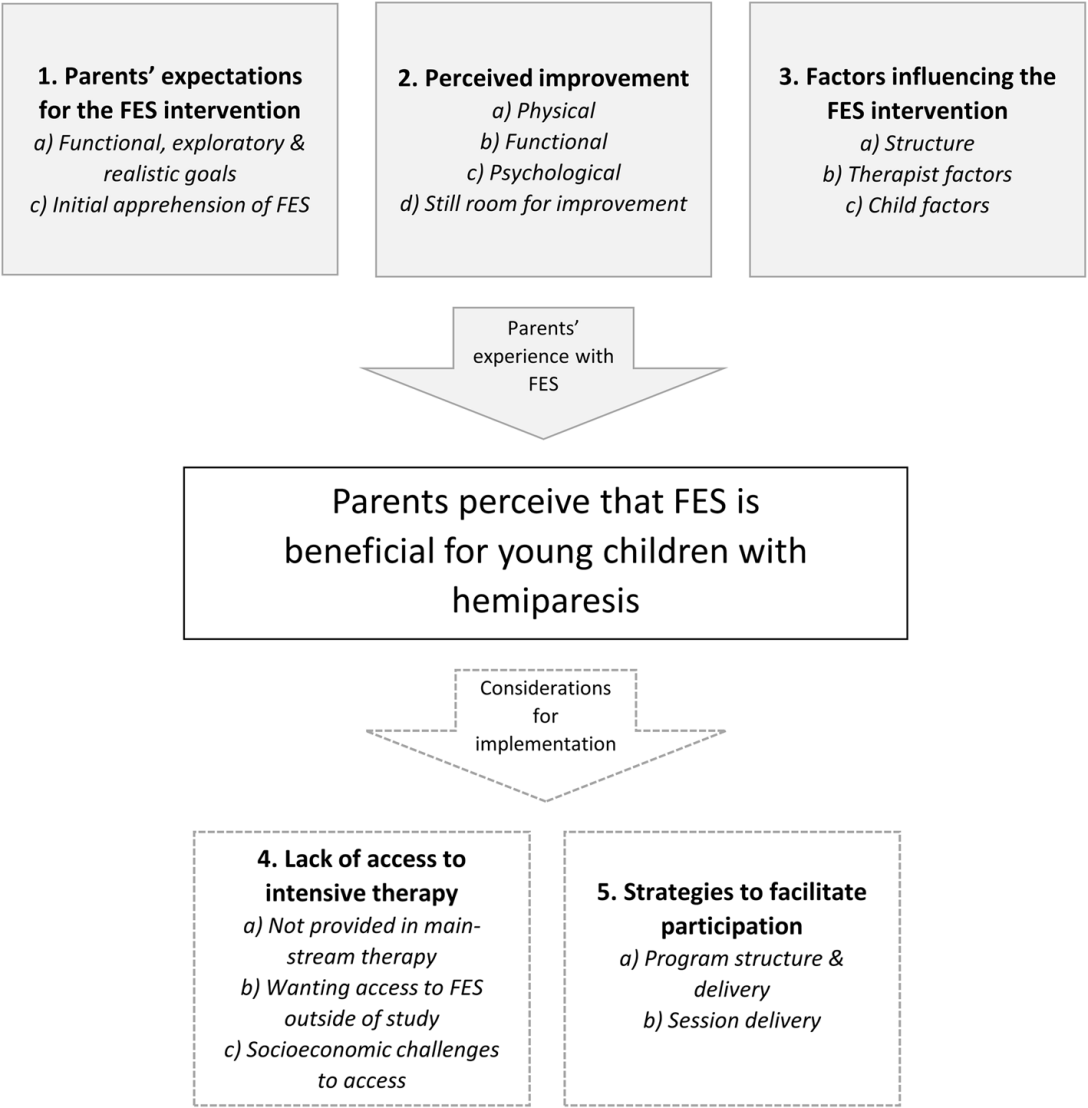
Diagrammatic representation of identified themes and sub-themes

Five themes concerning mothers’ perceptions of FES for children with hemiparesis were revealed; 1) Parents’ expectations for the FES intervention, 2) Perceived improvement, 3) Factors influencing the FES intervention, 4) Lack of access to intensive therapy, and 5) Strategies to facilitate participation. The themes and sub-themes are shown in Fig. 1 and Table 2.

#### 1. Parents’ expectations for the FES intervention

Parents’ expectations included a) functional, exploratory, and realistic goals, which they reported were largely achieved. However, some described feeling b) initial apprehension towards FES.

##### 1a. Functional, exploratory, and realistic goals

All participants mentioned functional goals such as dressing, feeding, and playing. Two participants mentioned an exploratory goal of participating “just to see if there’s other options and if they’re better than what he’s already doing” (P3), and to “[see] if this type of therapy [ …] would have a positive effect on him and the usage of his [affected] hand” (P10). When asked about the goals for participating in the study, one participant mentioned the value of exploring opportunities through research:

“You can’t really advance, you can’t make any intervention without research behind it … I wanted to be part of research that maybe can help some other kid … It’s not just for my kid. It’s also for future kids as well.” (P11).

A commonly shared view was that complete recovery of arm function and independence was not expected. P5 said “we were trying to be realistic in just thinking any use, any help she could give her [affected arm] would be ideal [ …] for anything, for eating, playing, anything.” Other participants made statements like “it’s never going to be perfect” (P4) or “I didn’t expect to see an overnight change or like some miracle cure” (P10). P8 discussed her lack of expectations, stating that “I don’t know where [he’s] going to be, and so you celebrate any little goal and achievement that comes along” (P8). Expectations for the retention of improvement following FES were also discussed. P4 said “I understand it’s not permanent, like you have to keep using the hand and using the arm and using the brain, and so, we’ll see, sort of, what the lasting impacts are.” Similarly, another participant was more focused on maintenance, saying that she wanted to keep her child “even at the same place where we’re at” (P6).

##### 1b. Initial apprehension towards FES

Several participants were concerned about their child’s tolerance for FES at the beginning of the intervention. Some participants indicated that they were “a little uncomfortable at first” (P4) and “kind of worried at the beginning” (P6) because they were unsure of how their child would react. Similarly, one mother was uncertain as she did not know “how [he] would tolerate it” (P10). Two participants expressed concerns about their child’s sensory challenges, including the mother whose child has a concomitant diagnosis of autism spectrum disorder: “I thought cause of his sensory issues as well there would be issues with the stickers” (P7).

#### 2. Perceived improvement

All participants noticed an improvement during the FES intervention phase that fell within three categories: a) physical, b) functional, and c) psychological improvement. However, the mothers indicated that there was d) still room for improvement.

##### 2a. Physical improvement

All participants noticed physical improvement in their child’s affected arm, with reduced tone and increased range of motion (ROM) most frequently reported. Participants described the arm and hand of their child to be “more open” (P3, P4, P5), “relaxed” (P5, P7), and that the arm “could fully extend” (P2). In comparison to the unaffected side, one participant stated that “when stretching [the child’s] arm out, it feels much more like the other hand” (P4). Improvements in awareness and sensation tolerance of the arm and hand were also observed, with one parent describing that prior to the intervention their child “wanted nothing to do with us touching [the arm]” and “now she’s giving us high fives with her [affected] hand” (P5).

While numerous improvements were noted, one mother noted that her child’s “shoulder got tighter [ …] she couldn’t actually reach above her head with a straight arm” (P6). However, while a decrease in ROM was noted at the shoulder, physical and functional improvements at the elbow and wrist were observed.

Participants reported varied rates of physical improvement. Some saw a difference in ROM or tone “within the first few weeks” (P4), or “within the first couple sessions” (P3). Two participants reported immediate change, stating “I noticed a difference [in elbow extension] right away” (P2) and “I could see [increased arm use] immediately after” (P10). Parents of children who participated in the control group prior to the FES intervention described that their child’s change in tone with FES “was more of an obvious change than it was with the control” (P3), while another described that “fine motor function was with FES, and the gross motor, like the bigger movements, was with control” (P8).

##### 2b. Functional improvement

Functional improvements in the areas of dressing, feeding, and/or play were noticed by all participants. A large component of this functional improvement related to bilateral use of the arms and hands, such as carrying the recycling bin (P7) or Lego container (P5), getting dressed (P4, P6), opening a chip bag or chocolate bar (P3), baking a cake (P2), reading (P11) and playing with a ball (P7, P8). Several participants compared their child’s function before and after the intervention, highlighting changes and new uses of the arm. P5 provides a notable example:

“Before, she would purely [ …] eat with her [unaffected] hand. And, it was never an attempt to introduce the [affected] hand on her own to try and eat. But, two weeks ago [ …] she was eating yogurt and just put the spoon in her [affected] hand on her own, I did not ask her, which I often do, but this day I did not [ …] she opened her [affected] hand, she put the spoon in by herself, held onto it, and brought it to her mouth countless times”.

Mothers often commented on the increased frequency and unprompted use of the affected arm, making statements like “she’s doing more without us having to remind her” (P2), and “(he’s) using it more spontaneously” (P7). One participant also noted changes in motor planning: “it’s not just the movement, it’s the planning of the movement as well that’s improved” (P8).

##### 2c. Psychological improvement

Half of the participants noticed improvements in their child’s pride and confidence following the FES intervention. The children were reportedly proud to demonstrate their new abilities, making comments like “look what I can do” (P2), “do you see what I’m doing?” (P5), and “see mom, I’m using my [affected] hand” (P4). Confidence was also associated with the acquisition of new skills, with one participant indicating that their child “feels very confident and empowered, because he feels that he has accomplished all this neat stuff” (P4), and another stating that “she maybe is more confident that she can do certain things if she uses both hands” (P5).

##### 2d. Still room for improvement

While all participants indicated that either their goals were achieved or that they were noticing progress, the majority indicated that there is still potential for further improvement. One participant mentioned that they could have worked a bit more on their child’s fingers (P2), another participant said that “I think she still has a ways to go” (P5), and another indicated that “[he] still struggles with dressing” (P8). However, there was general satisfaction with the improvements observed over the course of the intervention, with participants indicating that their child has “made leaps and bound of progress” (P5). Another participant articulated that while their child is “not there yet … I see it happening. I see like, potential for that … for it to be a lot easier now, because he has more functionality than he did before” (P4).

#### 3. Factors influencing the FES intervention

Three key factors were perceived to contribute to the success of the FES intervention: a) program structure, including flexibility and volume, b) therapist factors, and c) child factors.

##### 3a. Structure

Scheduling flexibility was the most frequently cited positive element of the structure of the FES program. Participants “thought it was great that [they] could do two sessions in one day” (P5), and two participants even said that they could not have participated in the study if it weren’t for the scheduling flexibility. Another positive aspect of the program was the volume and intensity of therapy provided. One participant felt that this volume was not available elsewhere: “it’s three times a week, like where are you going to get that much physio?” (P3). Participant 4 felt that “the intensity of it is probably one of the reasons that it’s working, and that it’s working so well” (P4).

The volume of the program was viewed as beneficial: “you’re getting maximum physio, right? Like, you’re getting lots and lots of therapy out of it” (P11). However, the number of weekly visits presented a challenge for many families. Participants shared that “it was a lot [ …] we thought twice about participating when we found out that it was three a week” (P4) and “I know it needed to be three a week, that was a little bit challenging” (P10). Some participants mentioned that transportation was difficult, saying “getting here and the drive” (P5) and “I just kind of wish there was something closer” (P3). When combining the volume of therapy and travel time, participants identified that “the biggest challenge is the time required, probably [ …] it’s long and it’s you know, well not quite every day, but it feels like every day” (P6). Despite the challenges, many participants indicated that the study was still worthwhile: “it was worth every hour, and every moment on the highway” (P8).

##### 3b. Therapist factors

**Flexibility and Individualization of Sessions**

The therapist’s flexibility and individualization of the session was frequently highlighted. Participants mentioned that the therapist was “good at asking what kind of things [he] likes to do, and you know, what he’s motivated by” (P4), “being flexible and changing activities as needed” (P5), and being “very receptive and accommodating to trying to get him to do what she wanted” (P10). These therapist qualities were believed to enable the continued engagement of the child.

**Rapport with Child**

Alongside flexibility and individualization of sessions, a positive relationship between the therapist and child was considered important. P7 described the therapist as “amazing”, stating that she “did a really good job and built rapport with him.” This participant went on to describe the therapist’s ability to balance acting with authority with being fun and engaging: “she [therapist] was the perfect balance of being firm enough cause [child] says no to everything, but … getting him engaged enough too” (P7). This opinion was shared by several participants, as they felt the therapist was “not too serious” (P11) and “the right balance of like sternness and fun” (P8).

##### 3c. Child factors

**General Disposition and Motivation**

Many participants made comments about their child’s resilience and motivation, saying that their “[child] is such a trooper” (P2), and “thank god like he’s a pretty motivated kid” (P3). Others discussed adaptability and patience, sharing that their child “adapts really well” (P7), or “is pretty patient” (P10). All these statements emphasize positive characteristics of the child’s disposition that may have contributed to the feasibility of the FES intervention.

Participants also recognized that children are not always motivated: “they’re kids too, right. So, sometimes they want to, and sometimes they don’t want to. You just gotta [ …] figure out how to get them to participate, because I know sometimes, she didn’t like it” (P2). Another factor that participants mentioned was the need for their child to become familiar with the setting and the therapist, noting that “a part of it … of him is getting comfortable with the person that he’s working with” (P3).

**Tolerance**

Participants reported that their children tolerated the stimulation to varying degrees. Most participants noted that the first few sessions were more challenging. For example, P5 said that “the first couple sessions were not terrible, but she just was kind of rattled, like didn’t know what to expect, didn’t know what was going on.” This mother also emphasized that she did not think her child was in pain, but rather was overwhelmed by the new sensation. P7 seemed to share this sentiment, saying that “it just felt weird [ …] he pulled away because it’s a new sensation.” Most participants said that their child became more comfortable with the feeling after a few sessions.

Some participants felt that tolerance was not an issue at all for their child. For example, one mother felt that their child’s “tolerance was pretty like high, he didn’t really complain” (P3), another said that their child was “very sensitive … but he’s handled it really well” (P4), and another said that once “we’d get there and we put them on and we play and she was fine” (P6). Interestingly, two participants reported variation in tolerance over time. P2 shared that initially the child “was able to tolerate it a lot more [ …] then, towards the end [of the intervention], she didn’t have as high of a tolerance” which they attributed to increased blood flow. Similarly, P10 indicated that over time “[he] got used to it and soldiered on,” but that “sometimes near the end [of the session], he was getting tired, he would start to try to pull at it [FES device]” (P10).

**Prior Experience with Therapy**

Most participants discussed their child’s previous involvement in therapy. Some speculated that heavy involvement in therapy may have influenced their child’s ability to engage in FES, as therapy is a regular component of their routine. For example, P2 said “like [her] schedule is super busy, like, she’s been doing therapy consistently… So, I think that will probably, you know, play a role as well.” Four families had previous experience with constraint induced movement therapy (CIMT), another form of intensive UE therapy.

#### 4. Lack of access to intensive therapy

Participants felt that there was a lack of access to intensive therapy, which was further described as a) not provided in mainstream therapy, b) wanting access to FES outside of the study, and c) socioeconomic challenges to access.

##### 4a. Not provided in mainstream therapy

Participants were disappointed by the lack of services available in the public system, making statements like “there aren’t services period … what there is, no one tells us about, we have to find out on our own” (P6) and “as he grows up, [therapy services] kind of started evaporating; they kind of just went away” (P11). Although three visits per week was often challenging, many participants were happy that they had the opportunity to complete this volume of therapy without a significant associated cost. These families “don’t get regular therapy unless [they’re] paying for it” (P6) and have communicated that “the higher functioning your child is, the less [the therapists] see them” (P3). Several participants compared their previous OT experience to FES. One participant said that “all our therapy up until now, until this, has been gross motor … we’ve never had OT like this before” (P5), and another shared that “the results are leaps ahead of what we would be able to accomplish with regular OT” (P4). Another mother emphasized that “this is the only therapy [child] has ever had on his hand” (P8). Following their experience with FES, participants questioned “why wouldn’t all kids have this? Like why wouldn’t everybody have an opportunity to access this if it’s going to help them?” (P4).

##### 4b. Wanting access to FES outside of the study

All participants expressed an interest in accessing FES upon completion of the study. Some were interested in the availability of a home device, while others were looking to incorporate FES into their treatment plans at private clinics. Despite the interest, some participants indicated that the availability of FES for children is limited. For example, P2 found a clinic closer to home that has an FES device, although when she “called and asked them if they’re doing it on kids and she’s like, “well, it’s in research”.” The lack of availability led to frustration: “This is what kills me. They have so many things for adults, and they have nothing for kids” (P2). Several participants expressed a desire to advocate for FES for other children, stating “I just think that everybody should have access to this, so I am happy to advocate for that to happen” (P4). Other participants shared that they “tell everybody [about FES]” (P8), and that “FES is included in every email I send to a brand new mom who’s just found out her kid has CP” (P6).

##### 4c. Socioeconomic challenges to accessing FES

Although all participants wanted to continue with FES, many recognized that completing this type and intensity of therapy may not be feasible for all families. As noted by one mother, “the frequency of it could be a bit overwhelming for a parent” (P11). Several shared that they were only able to attend every session thanks to family support, career flexibility, and access to a vehicle. Social class was also discussed, with one participant indicating that “upper-middle-class people who have those kind of luxuries … are probably more likely to be able to participate” (P4). Similarly, cost was discussed as a factor limiting access to FES programs, “these things are … expensive, and like not everyone’s insurance covers or they only cover some” (P3).

#### 5. Strategies to facilitate participation

Participants identified strategies related to either a) program structure and delivery or b) session delivery that could improve access and participation in FES programs or interventions (Table 3).

**Table 3:** Recommendations for Functional Electrical Stimulation (FES) with Young Children

Type	Perceived Challenge	Recommendations
Program structure & delivery	Difficulty balancing time commitment	• Ensure flexibility to accommodate family schedules (i.e. back to back sessions, intensive camps)
	Location of intervention	• Ensure FES offered in family friendly facilities to address sibling and family needs. • Improved availability in community settings would reduce travel time required for participation.
Session Delivery	Children unfamiliar with electrical stimulation	• Incorporate familiarization sessions to improve child comfort with the device and sensation. • Consider individual needs of each child to determine level of familiarization needed
	Engaging young children in intensive therapy	• Ensure therapist is flexible, creative, and provides an individualized approach to each session. • Child’s disposition and previous experience with therapy may influence engagement in therapy

##### 5a. Program structure and delivery

Due to the difficulties with location, the primary suggestions were either to provide FES as a home therapy or to make it available in the community closer to home. Half of the participants viewed home therapy as a good option, while the other half expressed concerns that children “work differently at home” (P2) and that balancing siblings would be challenging (P4). To offset the difficulty of commuting to a site three times a week for therapy, intensive camps were suggested (P3), the perceived feasibility of which was confirmed by other mothers. Two participants suggested that FES be offered in conjunction with other types of therapy, such as CIMT (P8, P10). Another suggestion was that the intervention be offered at a children’s facility to better address family needs, such as a play area for siblings (P8). Two parents mentioned that organizing extra assistance for transportation and childcare for siblings would be helpful.

##### 5b. Session delivery

Participants discussed several strategies to improve a child’s tolerance when receiving the stimulation. Some suggested distraction techniques, such as “do [ing] activities that are fun for the child, that kind of distract them” (P2), “chang[ing] the activity” when the child notices the stimulation. Others found it helpful to engage the child in the stimulation by allowing the child to “actually hold the machine and let [them] play around with it” (03), or taking “home a set of [electrodes] and practice” (05). Another strategy was to use the machine noises as an incentive, by telling the child “every time you do this, you’ll hear the beep and you get a point” [10]. Warm-ups and familiarization sessions were recommended to improve the child’s comfort with FES. Two mothers found it helpful when they did not attend the session, as their children responded better to the instructions from the trainers.

## Discussion

The results of this study indicate that mothers perceived FES to be a beneficial UE intervention for their children with unilateral CP. Improvements were reported across physical, functional, and psychological domains, suggesting that FES can have a widespread impact on a child’s well being. While parents reported goal achievement throughout the intervention, the consensus was that further improvement was possible. In fact, all parents were interested in continuing with FES after the study, however the lack of availability of this type of intensive therapy was frequently cited as a challenge. Other challenges were discussed, and suggestions for improving the access to and participation in FES emerged.

This is the first study to comprehensively explore parent perceptions of FES for their young children. Previous studies have collected parent perceptions of FES in children with CP, with Wright et al. reporting anecdotal comments [20] and Garzon et al. using a feedback questionnaire [21]. The use of semi-structured interviews in our study allowed for a greater depth of exploration of the thoughts and experiences of parents than the methodologies employed in the previously mentioned studies [27]. Despite the differing methodologies, our findings of improved functional and play-based UE use are consistent with those reported anecdotally [20]. Further, the challenge of time commitment required for the FES intervention was discussed by parents in both our study and Garzon et al.’s study [21].

A notable finding from this study relates to the reported improvements in spontaneous use of the affected arm. Children with unilateral CP often present with developmental disregard [30], and thus the increased spontaneous use following FES suggests that the intervention may help to overcome this impairment. CIMT in combination with bimanual training is an intervention that has been used to address developmental disregard in children with unilateral CP, although evidence supporting its effectiveness remains limited [31, 32]. FES may provide an alternative to CIMT for addressing developmental disregard in this population. However, since our findings are qualitative in nature, further research incorporating validated objective measures is required to quantify the effect of FES on development disregard [33].

Importantly, our study identified that FES is tolerable for young children with CP. This finding is particularly relevant due to the perception among some physical therapists that FES is not appropriate for children [22]. While children required some familiarization with the device and the sensation of FES, parents reported that their children adjusted to the stimulation and were able to successfully complete the intervention. Although tolerance was generally high, variation among tolerance levels both between children and from session to session were reported. This variation highlights the need to consider each child’s unique response to the stimulation, and the need for gradual familiarization and individualization of sessions. This is one of several recommendations for how to optimize the delivery of FES for young children (Table 3). Consideration of these recommendations will guide future research and implementation.

Parents’ involvement in advocating for FES and their interest in continuing with the therapy underscores the need to improve the availability of FES for children with unilateral CP. Since our intervention was conducted in a research setting, evaluating the use of FES in the community will be important to determine if and how FES is used in clinical practice. Exploring clinician’s perspectives of electrical stimulation would help to identify any barriers of use and maximize knowledge translation. Similarly, the child’s perspective on the use of FES for UE rehabilitation would be important to capture as a child’s view on their rehabilitation may not align with that of their parents’ [34] or their health care team [35]. Further, consideration of the challenges identified by parents such as cost, availability, and proximity of this therapy will be important to ensure equitable and effective implementation. Given the interest expressed from parents, these next steps will be instrumental in improving access to FES in the community.

### Limitations

Three interviews were conducted over the phone. As such, we were unable to capture non-verbal cues that may have added value to our data [36]. Additionally, since parents identified goals for the intervention and invested significant time and energy into participating in the study, they may have been subconsciously more observant of their child’s behaviours. Thus, their subjective reports of improved function may be a product of heightened awareness of these activities, rather than an indication of true improvement. Further, improvement in UE function can occur naturally with age. As such, it is essential that the parent perspectives of perceived improvement are compared to quantitative outcomes and to age-matched controls. Larger scale studies are needed to draw conclusions about the overall efficacy and required dosage of FES in young children with hemiparesis.

## Conclusion

Our findings indicate that FES is considered beneficial for children with unilateral CP from the perspective of parents. While the intervention did not restore full function in the affected UE, participants felt that their goals and expectations were largely met. Parents reported an absence of therapy programs in the community, FES specifically, targeting the UE for children with CP, which emphasizes the need for ongoing research to support the translation of this therapy into practice.

## Data Availability

Data cannot be shared publicly because data contain potentially identifying information. Data are available from the University Health Network Ethics Committee (contact via email: reb@uhnresearch.ca) for researchers who meet the criteria for access to confidential data.

## Abbreviations

FES: Functional electrical stimulation
UE: Upper extremity
CP: Cerebral palsy
